# A Temporal Threshold for Distinguishing Off-Wrist from Inactivity Periods: A Retrospective Actigraphy Analysis

**DOI:** 10.3390/clockssleep2040034

**Published:** 2020-11-12

**Authors:** Renske Lok, Jamie M. Zeitzer

**Affiliations:** 1 Department of Psychiatry and Behavioral Sciences, Stanford University, Stanford, CA 94305, USA; rlok@stanford.edu; 2 Mental Illness Research Education and Clinical Center, VA Palo Alto Health Care System, Palo Alto, CA 94304, USA

**Keywords:** actigraphy, accelerometry, circadian, inactivity, off-wrist, sleep, temporal threshold

## Abstract

(1) Background. To facilitate accurate actigraphy data analysis, inactive periods have to be distinguished from periods during which the device is not being worn. The current analysis investigates the degree to which off-wrist and inactive periods can be automatically identified. (2) Methods. In total, 125 actigraphy records were manually scored for ‘off-wrist’ and ‘inactivity’ (99 collected with the Motionlogger AMI, 26 (sampling frequency of 60 (*n* = 20) and 120 (*n* = 6) s) with the Philips Actiwatch 2.) Data were plotted with cumulative frequency percentage and analyzed with receiver operating characteristic curves. To confirm findings, the thresholds determined in a subset of the Motionlogger dataset (*n* = 74) were tested in the remaining dataset (*n* = 25). (3) Results. Inactivity data lasted shorter than off-wrist periods, with 95% of inactive events being shorter than 11 min (Motionlogger), 20 min (Actiwatch 2; 60 s epochs) or 30 min (Actiwatch 2; 120 s epochs), correctly identifying 35, 92 or 66% of the off-wrist periods. The optimal accurate detection of both inactive and off-wrist periods for the Motionlogger was 3 min (Youden’s Index (*J*) = 0.37), while it was 18 (*J* = 0.89) and 16 min (*J* = 0.81) for the Actiwatch 2 (60 and 120 s epochs, respectively). The thresholds as determined in the subset of the Motionlogger dataset showed similar results in the remaining dataset. (4) Conclusion. Off-wrist periods can be automatically identified from inactivity data based on a temporal threshold. Depending on the goal of the analysis, a threshold can be chosen to favor inactivity data’s inclusion or accurate off-wrist detection.

## 1. Introduction

Actigraphy, the use of wrist-worn tri-axial accelerometers to track movement patterns, is a technology that has been used for over 25 years in clinical sleep research [1]. Actigraphic data can be used to impute sleep and wake states, and are most valid when detecting waking up during sleep using algorithms that are variations on threshold moving averages (i.e., when the activity is of sufficient length or duration at night to indicate an awakening) [1,2]. While actigraphy is not a perfect representation of sleep [3], and can both over- and underestimate sleep depending on the circumstances [4], it has several advantages over polysomnography, the criterion standard for the determination of sleep and wakefulness. Notably, as compared to polysomnography, actigraphy is less invasive, less expensive, more amenable to long duration (weeks to months) recording, and can yield insight into daytime behavior.

Given the advantages of actigraphy, these devices have been used in several large studies to examine longitudinal changes in behavior (e.g., UK Biobank [5], Osteoporotic Fractures in Men [6], Study of Osteoporotic Fractures [7]). As actigraphs are marketed as water-resistant or waterproof, most studies direct participants to continuously wear the actigraph, including during periods of bathing, swimming, or washing dishes. Despite these instructions, many individuals will remove the actigraph for intermittent durations and at random times. This becomes problematic as most of the available actigraphs do not have automatic off-wrist detection, and off-wrist periods would be automatically scored as continuous episodes of “sleep” due to the prolonged lack of activity. As such, it has been proposed that actigraphic records should be manually inspected to exclude off-wrist periods [8,9,10]. These periods are visually distinct from sleep as they have no movement, while sleep is characterized by sporadic, brief periods of movement. While manual inspection is appropriate and useful in smaller data sets, the ability to collect weeks of actigraphy data from thousands of individuals precludes this type of hand-curation. To facilitate the accurate actigraphy data analysis of large data sets, the goal of this study was to investigate the degree to which off-wrist and inactive periods could be automatically identified. As off-wrist periods are typically longer than inactive periods, our a priori assumption was that a fixed duration threshold could adequately discriminate inactive and off-wrist periods. Given the different collection frequencies that are often used for actigraphy data, we also examined the impact of collection frequency, as well as the use of different devices, on the performance of automated off-wrist detection.

## 2. Results

We examined records from 125 individuals who wore one of three different devices for approximately two weeks (Table 1). Our participants were young (mean ± sd; 26.5 ± 5.32) and mostly male (61.1%). Out of 125 data files, 74.4% (93 files) contained off-wrist data, as identified by hand curation. From the Motionlogger AMI, 71.7% (71 files) contained off-wrist periods. For the Actiwatch 2, 88.5% of the files contained off-wrist data (all of the 120 s epoch data, 16 of the 60 s epoch data). Further specifications of inactivity and off-wrist periods can be found in Table 1.

**Figure 1:**
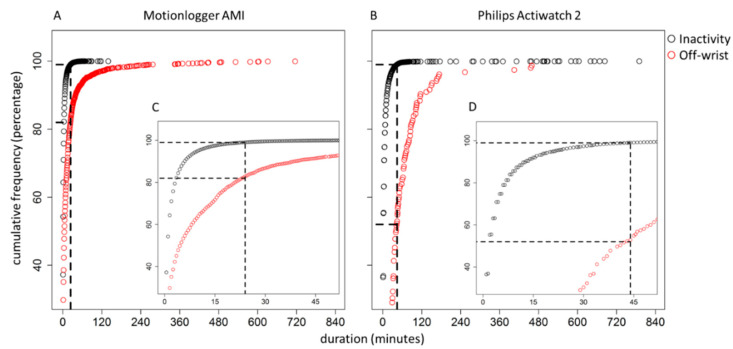
Cumulative frequency plots indicating the duration of consecutive strings of data marked as ‘inactivity’ or ‘off-wrist’. Both the complete distribution of inactivity (**A**,**B**) and off-wrist periods (**C**,**D**) for the Motionlogger and Actiwatch 2 are presented. Black circles represent data marked as inactivity, while red dots indicate off-wrist periods. Vertical dotted black lines indicate 99% correctly identified inactivity data of the Motionlogger and Actiwatch. Data left of the vertical line indicate inactive data correctly identified as such and off-wrist data incorrectly identified as inactivity. Data to the right of the dotted lines indicate inactivity data incorrectly identified as off-wrist, and off-wrist data correctly identified as such.

On both devices, the lengths of inactive events, defined as episodes of consecutive zeroes that are not considered off-wrist periods, were shorter compared to off-wrist periods (Table 1, Figure 1). For example, as is visualized in Figure 1, 99% of inactive events are shorter than 24 (Motionlogger) or 44 min (Actiwatch 2). These same thresholds would capture only 83% (Motionlogger) and 48% (Actiwatch 2) of the off-wrist periods. To confirm the initial findings, the divided Motionlogger dataset revealed similar thresholds—99% of inactivity data were shorter than 24 min (training), which would capture only 83% of the off-wrist periods. The thresholds as determined in the training dataset would result in capturing 98% of inactivity data and 81% of off-wrist periods in the testing dataset.

**Figure 2:**
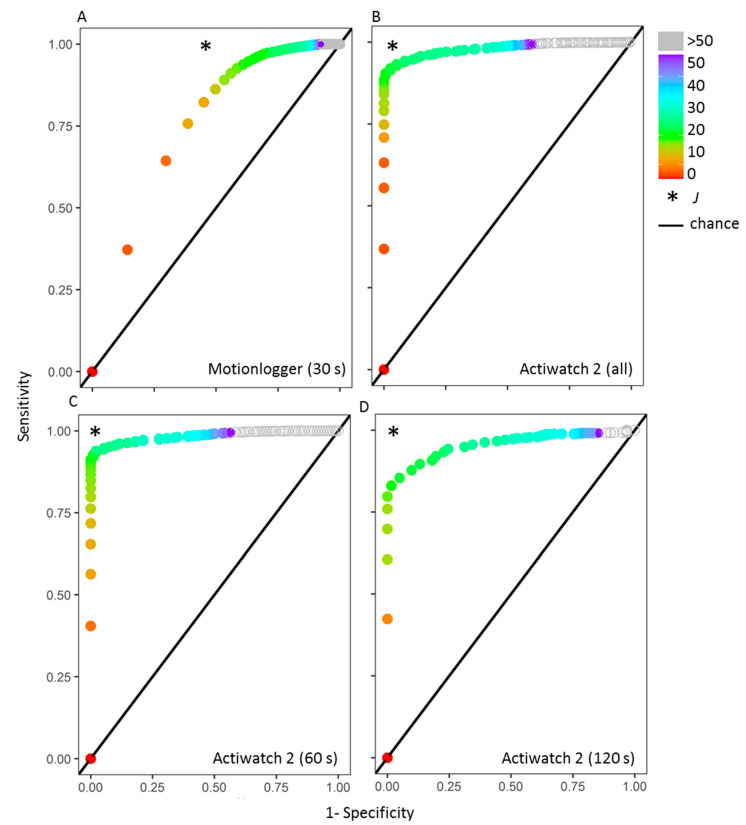
ROC analysis of correctly identified off-wrist and inactivity periods. Data are plotted as 1-specificity (inverse of the classification accuracy of off-wrist) versus sensitivity (classification accuracy of inactive) with each point representing a different threshold (color-coded). Data are shown for the Motionlogger (**A**) and Actiwatch 2 (**B**), as well as for the separate collection frequencies of the Actiwatch 2—60 s (**C**) and 120 s epochs (**D**).

To compare the performances of different thresholds for discriminating off-wrist from inactive, we used ROC analysis. True positives were defined as inactive periods correctly identified as inactive, while true negatives were defined as off-wrist periods correctly identified as off-wrist. These are plotted for different threshold lengths (Figure 2). The optimal threshold (i.e., one equally balancing the accurate detection of both inactive and off-wrist) for the Motionlogger was 3 min (Youden’s Index (*J*) = 0.37), while the optimal threshold for the Actiwatch 2 was 14 min (*J* = 0.89). There were similar optimal thresholds for the train (3 min, *J* = 0.37) and test (4 min, *J* = 0.43) datasets as for the two different interval collection periods in the Actiwatch 2 data—18 min (60 s intervals, *J* = 0.92) and 16 min (120 s intervals, *J* = 0.81).

One could argue, however, that it is more important to minimize the misclassification of intervals of inactivity as off-wrist, as such a misclassification would lead to either the exclusion or imputation of valid data. Therefore, we also calculated the optimum threshold based on the maximization of sensitivity (Table 2). The greater capture of all of the inactive episodes (i.e., higher sensitivity) results in a larger cut-off point, which increases the number of off-wrist periods misclassified as inactive (i.e., lower specificity).

## 3. Discussion

Using a temporal threshold, periods during which an actigraph is not worn can be discriminated from periods when individuals are wearing the actigraph but are in a quiescent state. The accuracy of this discrimination appears to be dependent on the collection interval of the actigraph. Using a 30 s data bin (Motionlogger), a threshold of 11 min accurately classified 95% of the inactive data (i.e., less than 5% of the inactive data are longer than 11 min) and 35% of the off-wrist data (i.e., 65% of the off-wrist data are shorter than 11 min). This was confirmed by analysis conducted on the training and testing datasets. With a 60 s data bin (Actiwatch 2), a threshold of 20 min accurately classified 95% of the inactive data and 92% of the off-wrist data. With a 120 s data bin (Actiwatch 2), a threshold of 30 min accurately classified 95% of the inactive data and 66% of the off-wrist data. Moving the threshold to longer durations increases the capture of more of the inactive data, but at a cost of losing the accurate classification of off-wrist periods. For example, moving the Motionlogger (30 s bin) threshold to 24 min will capture an additional 4% of the inactive data, but at a cost of the misclassification of 18% more of the off-wrist data. As such, our suggestion is to use the 95% accuracy thresholds to delineate data as off-wrist. There are many off-wrist periods lasting shorter periods than these thresholds, but these are not discriminable from inactive periods through the use of a threshold alone. Whether imputation of these short periods impacts actigraphy-based metrics of sleep and circadian rhythms remains open for further study.

The current analyses focus on inactive periods, defined as a consecutive string of zeroes. The presence of an activity count higher than zero does not necessitate wakefulness [11], nor is a consecutive string of zeroes necessarily sleep. While many articles have been written on the best way to impute sleep from movement [12], this is not the goal of the current analysis. The inactive data that we identified in these actigraph records occur at all times of day and night. The studies in which these individuals participated had a common set of instructions to maintain a regular sleep schedule at night. It would, therefore, be likely that most of the inactive periods occurring at night are consistent with sleep. The inactive periods during the daytime or evening, however, could be brief naps or periods of limited physical behavior (e.g., sitting on a couch). Whether these inactive periods represent sleep or quiescent behavior, however, is not relevant to the current analysis, which was focused on separating valid data from data collected during times at which the device was not being worn.

There were many more inactive events of similar length recorded by the Motionlogger than Actiwatch 2. There were also many more off-wrist events recorded by the Motionlogger, but these were typically shorter than those recorded by the Actiwatch 2. Since we were not able to examine the same recording frequencies on both devices, we cannot determine the exact cause of these differences, but they are most likely due to differences in the sensitivity of the devices and differences in the collection frequency (Table 1). Future studies should consider whether both or either of these two variables have a significant impact on differentiating off-wrist from inactive periods.

Another limitation of this study is that we examined healthy, young individuals with generally good sleep quality. We cannot conclude that the use of a threshold developed in this population would provide universally similar results in different populations, such as individuals with insomnia, older individuals, or individuals with musculoskeletal issues. Further studies in these types of specific populations are necessary. Our study does indicate, however, that in healthy young individuals, off-wrist periods can be separated from inactivity data, based on a simple temporal threshold. Higher thresholds result in greater accuracy in terms of capturing inactive periods, but will miss many off-wrist periods. Depending on the goal of the analysis, a threshold can be chosen to favor inactivity inclusion or accurate off-wrist detection. In cases in which hand scoring is not feasible, to increase automated scoring accuracy, this analysis could be applied to datasets as a pre-processing step.

## 4. Materials and Methods

We examined actigraphy data from 125 healthy, young individuals who had participated in a variety of in-laboratory protocols at different times of year. All participants had signed informed consent prior to any experimental procedures. All methods conform to the principals laid out in the Declaration of Helsinki. Actigraphy data were collected at home and with the instructions that individuals should wear their actigraph continuously for at least seven days and maintain a regular sleep–wake schedule. These data were collected with two different devices, the Motionlogger (*n* = 99, Ambulatory Monitoring Inc., Ardsley, NY, USA) and Actiwatch 2 (*n* = 26, Philips, Bend, OR, USA). Data were collected in 30 s epochs with the Motionlogger and at two different epoch lengths with the Actiwatch 2 (60 s epochs, *n* = 20; 120 s epochs, *n* = 6). During screening, all participants also completed the Pittsburgh Sleep Quality Index (PSQI); the entry criterion was the absence of significant sleep disruption (PSQI < 5).

**Figure 3:**
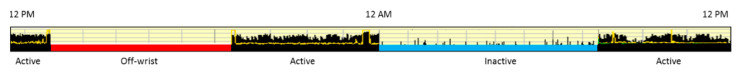
Example of off-wrist, active and inactive data. Movement data (arbitrary units) are plotted as vertical black lines over time (x-axis); light data are shown as a yellow line. Off-wrist periods (red horizontal bar) are characterized by the absence of activity bouts for prolonged periods of time (with or without light information). Active periods (black horizontal bar) are visible as high-density, high- or low-activity bouts (usually with elevated light levels). Inactive periods (blue horizontal bar) are represented by low-density, low-activity bouts (and usually an absence of light).

To mark off-wrist and inactivity periods, ActionW (Motionlogger; v.2.7, Ambulatory Monitoring Inc, Ardsley, NY, USA) and Actiware (Actiwatch 2; v.5, Respironics, Bend, OR, USA) were used to visualize data. Off-wrist periods were visually defined by a sudden drop in activity to zero, followed by prolonged periods of zero activity counts without any activity bouts (Figure 3). Periods of inactivity were marked by a sudden drop in activity, followed by periods in which activity counts of zero were interrupted by low-density, low-activity bouts, and concluded by a steep increase in activity (Figure 3). Light data (wrist-recorded illumination in lux) were used as supplemental information, as these data did not consistently discriminate inactivity from off-wrist periods. The sleep-detection algorithms are insufficient to discriminate inactivity from sleep and, as such, we chose to not delineate our inactive periods into the categories of “sleep,” “nap,” or “inactive”. Actigraphy data were manually scored epoch-by-epoch on an individual level for all recorded days.

R-studio (version 3.6.0, Rstudio, Boston, MA, USA) was used for data analysis. For automated data processing, the length of all strings of consecutive bouts in which activity = 0 were calculated. For example, if there were 15 consecutive 30 s epochs in which activity = 0, this would be counted as one event with a length of 7.5 min. Events were further defined as being valid or “off-wrist” by visual marking. Data were both plotted with cumulative frequency percentage and analyzed with receiver operating characteristic (ROC) curves. The performance of ROC curves was determined by calculating Youden’s Index (sensitivity + specificity − 1), such that sensitivity is determined by true positives (inactive accurately classified as inactive) divided by all positives (number of episodes below that threshold) and specificity is true negatives (off-wrist accurately classified as off-wrist) divided by all negatives (number of episodes above that threshold) [13]. Data for the ROC analyses were calculated within participants and then averaged across participants. To validate findings, the complete analysis was repeated on a training subset (74.7%, *n* = 74) of the Motionlogger dataset (*n* = 99). Thresholds determined in this training subset were tested in the remaining dataset (testing, 25.3%, *n* = 25).

## Figures and Tables

**Table 1:** Overview of inactive and off-wrist parameters from the Motionlogger and Actiwatch 2 devices. IQR: interquartile range, SD: standard deviation.

Collection Device	Sensitivity (g-Force)	*n*	Collection Frequency	Hand Curated Parameter	Number of Days of Collection (Mean ± SD)	Number of Events per Person (Mean ± SD)	Length of Event (min) (Median (IQR))
Motionlogger (all)	0.01	99	30 s	Inactive	13.6 ± 1.73	1979 ± 517.0	2.0 (1.0–5.0)
				Off-wrist		28.35 ± 28.63	10 (3.0–34)
Motionlogger (training)	0.01	74		Inactive	13.5 ± 1.80	1999 ± 514.6	2.0 (1.0–5.0)
				Off-wrist		29.94 ± 30.25	9 (3.0–33)
Motionlogger (testing)	0.01	25		Inactive	13.8 ± 1.53	1906.8 ± 520.1	2.0 (1.0–6.0)
				Off-wrist		23.000 ± 21.11	14 (3.0–37)
Actiwatch 2	0.05	20	60 s	Inactive	13.5 ± 1.88	1275 ± 397.5	2.0 (1.0–5.0)
				Off-wrist		5.875 ± 3.862	44 (29–84)
Actiwatch 2		6	120 s	Inactive	17.2 ± 6.42	650.0 ± 132.7	2.0 (1.0–4.0)
				Off-wrist		10.16 ± 9.474	19 (14–33)

**Table 2:** Optimizing inclusion of inactive data and impact on off-wrist detection.

Device	Collection Interval	Fixed Sensitivity	Specificity	Cut-Point
Motionlogger (all)	30 s	99%	17%	24 min
Motionlogger (training)			17%	2 min
Motionlogger (testing)		98%	19%	
Actigraph 2	60 s	99%	51%	42 min
Actigraph 2	120 s		33%	70 min
Motionlogger (all)	30 s	95%	35%	11 min
Motionlogger (training)			35%	10 min
Motionlogger (testing)		94%	42%	
Actigraph 2	60 s	95%	92%	20 min
Actigraph 2	120 s		66%	30 min
